# A Cytotoxic Type III Secretion Effector of *Vibrio parahaemolyticus* Targets Vacuolar H^+^-ATPase Subunit c and Ruptures Host Cell Lysosomes

**DOI:** 10.1371/journal.ppat.1002803

**Published:** 2012-07-19

**Authors:** Shigeaki Matsuda, Natsumi Okada, Toshio Kodama, Takeshi Honda, Tetsuya Iida

**Affiliations:** 1 Laboratory of Genomic Research on Pathogenic Bacteria, International Research Center for Infectious Diseases, Research Institute for Microbial Diseases, Osaka University, Suita, Osaka, Japan; 2 Department of Bacterial Infections, Research Institute for Microbial Diseases, Osaka University, Suita, Osaka, Japan; Duke University, United States of America

## Abstract

*Vibrio parahaemolyticus* is one of the human pathogenic vibrios. During the infection of mammalian cells, this pathogen exhibits cytotoxicity that is dependent on its type III secretion system (T3SS1). VepA, an effector protein secreted via the T3SS1, plays a major role in the T3SS1-dependent cytotoxicity of *V. parahaemolyticus*. However, the mechanism by which VepA is involved in T3SS1-dependent cytotoxicity is unknown. Here, we found that protein transfection of VepA into HeLa cells resulted in cell death, indicating that VepA alone is cytotoxic. The ectopic expression of VepA in yeast *Saccharomyces cerevisiae* interferes with yeast growth, indicating that VepA is also toxic in yeast. A yeast genome-wide screen identified the yeast gene *VMA3* as essential for the growth inhibition of yeast by VepA. Although *VMA3* encodes subunit c of the vacuolar H^+^-ATPase (V-ATPase), the toxicity of VepA was independent of the function of V-ATPases. In HeLa cells, knockdown of V-ATPase subunit c decreased VepA-mediated cytotoxicity. We also demonstrated that VepA interacted with V-ATPase subunit c, whereas a carboxyl-terminally truncated mutant of VepA (VepAΔC), which does not show toxicity, did not. During infection, lysosomal contents leaked into the cytosol, revealing that lysosomal membrane permeabilization occurred prior to cell lysis. In a cell-free system, VepA was sufficient to induce the release of cathepsin D from isolated lysosomes. Therefore, our data suggest that the bacterial effector VepA targets subunit c of V-ATPase and induces the rupture of host cell lysosomes and subsequent cell death.

## Author Summary

*Vibrio parahaemolyticus* is a bacterial pathogen that causes food-borne gastroenteritis and also wound infection and septicemia. It exhibits cytotoxicity that is dependent on its type III secretion system (T3SS1) during the infection of mammalian cells. Although an effector VepA plays a major role in the cytotoxicity, the mechanism was unknown. Here we show that VepA targets subunit c of the vacuolar H^+^-ATPase (V-ATPase) and induces the rupture of host cell lysosomes. We found that VepA alone is cytotoxic in HeLa cells and also toxic in yeast *Saccharomyces cerevisiae*. Using a yeast genome-wide screening, we identified yeast V-ATPase subunit c as essential for the toxicity of VepA to yeast. We also demonstrated that knockdown of V-ATPase subunit c decreased VepA-mediated cytotoxicity toward HeLa cells and that VepA interacted with subunit c of V-ATPase. During infection, lysosomal contents leaked into the cytosol prior to cell lysis, and VepA was necessary and sufficient for this leakage. Our data suggest that a bacterial effector VepA ruptures lysosomes, the “suicide bags” of host cells, by targeting the evolutionarily conserved V-ATPase, and elicits subsequent cell death.

## Introduction

*Vibrio parahaemolyticus*, a Gram-negative marine bacterium, is a major food-borne pathogen that causes acute human gastroenteritis associated with the consumption of seafood. This pathogen also causes wound infections and septicemia in humans [1]–[3]. *V. parahaemolyticus* possesses virulence factors such as thermostable direct hemolysin (TDH) and two separate type III secretion systems (T3SSs), namely, T3SS1 and T3SS2 [4], [5]. T3SSs are protein export systems that enable bacteria to secrete and translocate proteins known as effectors into the cytoplasm of host cells. Translocated effectors modify host cell function and allow pathogens to promote infection and cause disease [6], [7]. *V. parahaemolyticus* T3SS1 is involved in the cytotoxicity to various mammalian cells, whereas T3SS2 is related to the enterotoxicity of this organism [8]–[10]. T3SS1-induced cell death occurs rapidly, within several hours after the inoculation of *V. parahaemolyticus*; is independent of caspases; and is associated with autophagy [11], [12]. The transcription of the T3SS1 genes of *V. parahaemolyticus* is regulated by a dual regulatory system consisting of the ExsACDE regulatory cascade and H-NS [13]. To date, VepA (VP1680/VopQ), VopS (VP1686) and VPA450 have been identified as T3SS1 effectors [14]–[18]. VopS functions as an AMPylator and contributes to cell rounding [17]. VPA450 acts as an inositol phosphatase and induces plasma membrane blebbing [18]. It has previously been shown that the deletion of *vopS* or *vpa450* has little effect on T3SS1-dependent acute cytotoxicity, but a mutant strain of *V. parahaemolyticus* in which *vepA* was deleted (Δ*vepA*) lost its cytotoxicity, suggesting that VepA plays a major role in T3SS1-dependent cytotoxicity [14]–[16].

VepA is a 50-kDa protein and consists of 492 amino acids. Although VepA has no homology to known proteins, the amino-terminal 100 amino acids of VepA are required for secretion by T3SS1 [15]. VepA has also been reported to be involved in the activation of autophagy and mitogen-activated protein kinases (MAPKs) [16], [19]. However, the mechanism by which VepA is involved in acute cytotoxicity in host cells is unclear. To understand the mechanism underlying the T3SS1-dependent cytotoxicity of *V. parahaemolyticus*, elucidation of the function of VepA within host cells is important because VepA appears to play a critical role in cytotoxicity. In this study, we showed that VepA itself is a cytotoxic effector, and we screened for host factors essential for the cytotoxicity of VepA using yeast genome-wide analysis to elucidate the function of VepA.

## Results

### VepA, a T3SS1-effector, itself is cytotoxic

**Figure 1:**
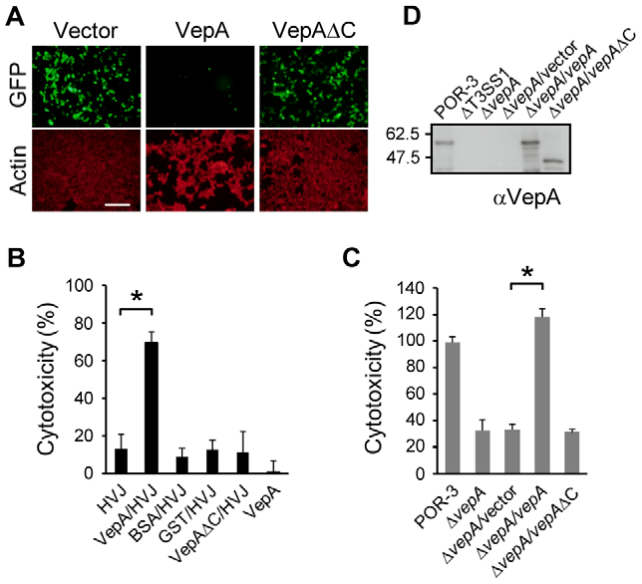
VepA is cytotoxic only inside cells. (A) Representative images of 293T cells transfected with GFP-fused VepA or VepAΔC. Actin was stained with rhodamine-phalloidin to visualize the cells. Scale bar, 200 µm. (B) The indicated proteins were delivered into HeLa cells using the HVJ envelope, or HeLa cells were treated with 100 µg ml^−1^ VepA without HVJ; cytotoxicity was determined after 4 h. The values represent the mean ± SD for a minimum of three independent experiments. (C) HeLa cells were infected with the indicated strains for 4 h and analyzed for lactate dehydrogenase (LDH) release as a measure of cytotoxicity. *P<0.01. (D) Secretion of VepA or VepAΔC. The indicated strains were grown in LB broth for 6 h, and the secreted proteins were subjected to immunoblot analysis using an anti-VepA antibody. The migration positions of the molecular weight markers are indicated on the left side of the panel.

To understand the function of VepA, we first examined the expression of green fluorescent protein (GFP) in 293T cells transfected with pEGFP-VepA. However, GFP-VepA expression was not detected (Figure 1A), raising the possibility that VepA itself is quite toxic in cells. To evaluate whether VepA itself is cytotoxic, we introduced purified VepA into HeLa cells by protein transfection using the hemagglutinating virus of Japan (HVJ) envelope vector. The delivery of VepA into cells caused a significant decrease in cell viability, in contrast to the delivery of glutathione-*S*-transferase (GST), bovine serum albumin (BSA) or HVJ alone (Figure 1B). VepA did not affect cell viability in the absence of the HVJ vector. These results suggest that VepA itself is cytotoxic and effective only inside cells, not outside cells. By contrast, the delivery of a truncated VepA protein lacking the C-terminal 92 amino acids (1–400, VepAΔC) into HeLa cells did not affect the viability of the cells (Figure 1B). Although complementation of the wild-type *vepA* gene in the Δ*vepA* strain (POR-3Δ*vepA*/*vepA*) rescued the infection-mediated cytotoxicity, complementation with *vepA*ΔC (POR-3Δ*vepA*/*vepA*ΔC) did not (Figure 1C). We also confirmed that VepAΔC is secreted from POR-3Δ*vepA*/*vepA*ΔC (Figure 1D), excluding the possibility that VepAΔC is not expressed or not secreted in *V. parahaemolyticus*. GFP-VepAΔC was successfully expressed in 293T cells, in contrast to GFP-VepA (Figure 1A), suggesting that VepAΔC is stable in cells. Taken together, these results indicate that VepAΔC loses the cytotoxicity.

To investigate the mechanism underlying VepA-dependent cytotoxicity, we tested whether autophagy and the MAPK signaling pathway were required for the cytotoxicity induced by *V. parahaemolyticus*. Inhibiting autophagy or MAPK with short interfering RNAs (siRNAs) or inhibitors did not affect the cytotoxicity (Figure S1), indicating that the contributions of autophagy and the MAPK signaling pathway to cytotoxicity are negligible. The cell-permeable pan-caspase inhibitor carbobenzoxy-valyl-alanyl-aspartyl-[*O*-methyl]-fluoromethylketone (Z-VAD-FMK) also did not affect the cytotoxicity, a result that is consistent with those of a previous study which the *V. parahaemolyticus* strain NY-4 was used [12].

### Yeast genome-wide screening identified a candidate as the target of VepA

**Figure 2:**
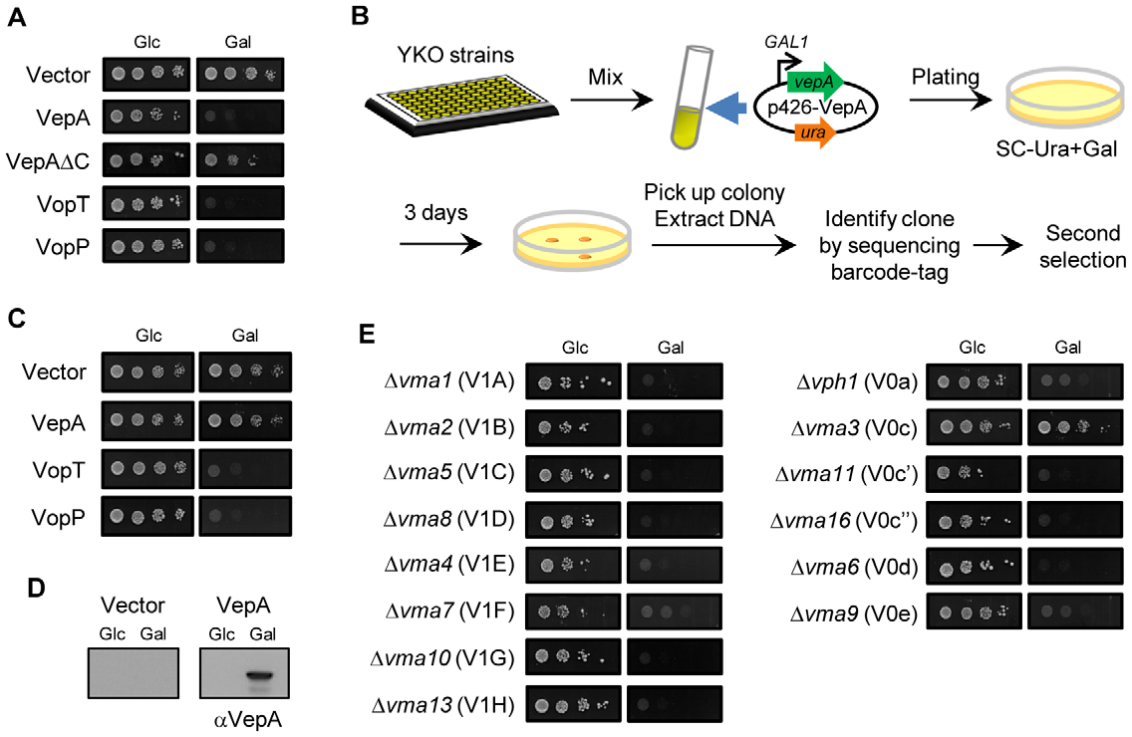
Subunit c of V-ATPase is involved in the toxicity of VepA to yeast. (A) *S. cerevisiae* BY4730 was transformed with the vector p426 or plasmids encoding wild-type VepA or VepAΔC. Then, 10-fold serially diluted cultures of yeast harboring each plasmid were spotted onto glucose (Glc) or galactose (Gal) plates and incubated for 72 h. (B) Scheme of the genome-wide screen for yeast knockout (YKO) strains resistant to VepA. YKO strains were grown individually in YPD broth in 96-well plates. For each plate, the strains were grown, pooled into a single culture and transformed with p426-VepA. The transformants were plated on SC plates lacking uracil and containing galactose (SC-Ura+Gal). After incubation at 30°C for 3 days, isolated colonies were analyzed to identify strains as described in Materials and Methods. (C) The growth patterns of the 10-fold serial dilutions of the Δ*vma3* strains harboring p426, p426-VepA, p426-VopT or p426-VopP on Glc and Gal plates are shown. (D) Immunoblot analysis using an anti-VepA antibody against lysates from Δ*vma3* strains harboring p426 or p426-VepA grown in Glc or Gal. (E) The sensitivity of V-ATPase mutants to VepA. A total of 14 yeast V-ATPase mutants (V_1_ domain; A, B, C, D, E, F, G, H, V_0_ domain; a, c, c′, c″, d, e) were transformed with p426-VepA. Then 10-fold dilution cultures of each mutant harboring p426-VepA were spotted onto SC-Ura+Glc or SC-Ura+Gal plates and incubated for 3 days at 30°C.

*Saccharomyces cerevisiae* has been widely used as a model system to study eukaryotic cells. An increasing number of reports have shown that the expression of bacterial effectors inhibits yeast growth, and this inhibition is implicated in the activity of effectors that affect cellular processes conserved among eukaryotic cells [20]. To determine whether VepA can inhibit yeast growth, we transformed *S. cerevisiae* BY4730 with the p426 expression plasmid encoding VepA and expressed VepA under the control of the *GAL1* promoter. The ectopic expression of VepA inhibited the growth of yeast (Figure 2A), indicating that VepA is also toxic in yeast. By contrast, VepAΔC, which lacks cytotoxicity (Figure 1A, 1B and 1C), was less toxic in yeast, suggesting a correlation between the cytotoxic effects of VepA in HeLa cells and its toxicity in yeast.

Next, we used a yeast knockout (YKO) strain library [21] to screen for host genes that are essential for the toxicity of VepA (Figure 2B). To express VepA in non-essential gene mutant yeast strains and screen for clones that are able to grow in the presence of VepA, we transformed 56 pools of YKO strains (one pool typically includes 95 strains) with the p426 expression plasmid encoding VepA and plated the yeast onto SC plates lacking uracil and containing galactose (SC-Ura+Gal). To examine the plating efficiency, the transformants were also plated onto SC plates lacking uracil and containing glucose (SC-Ura+Glc), yielding at least 1,000 colonies (giving >10×coverage). Using this genome-wide screen, we found that the Δ*vma3* strain was insensitive to the toxicity of VepA (Figure 2C). No MAPK- or autophagy-related gene mutants were identified in this screen, a result that is consistent with the cytotoxicity analysis presented in Figure S1. The expression of VepA in the Δ*vma3* strain was confirmed under inducing conditions (Figure 2D). The ectopic expression of VopT and VopP, which have been reported to inhibit yeast growth [9] (Figure 2A), was toxic to the Δ*vma3* strain (Figure 2C), thus excluding the possibility that the Δ*vma3* strain was non-specifically insensitive to bacterial effectors. Complementation of the *VMA3* gene in the Δ*vma3* strain restored the susceptibility to the toxicity of VepA (Figure S2A). *VMA3* encodes subunit c of V-ATPase. V-ATPases, which are highly conserved in eukaryotic cells, are composed of two domains: a peripheral, catalytic V_1_ domain and an integral V_0_ domain that serves as the basal body. V-ATPases serve as proton pumps to acidify intracellular compartments [22]. In yeast, the V_1_ and V_0_ domains contain eight (A, B, C, D, E, F, G and H) and six (a, c, c′, c″, d and e) subunits, respectively. Because the deletion of any subunit causes a functional deficiency in V-ATPases [23], we determined whether the function of the V-ATPase is involved in the VepA-mediated growth defects in yeast. VepA was expressed in the yeast strains containing mutations of each component of the V-ATPase, and their growth was observed. With the exception of Δ*vma3*, none of the yeast strains with mutations in the V-ATPase subunits were able to grow in the presence of VepA (Figure 2E), thus indicating that the function of the V-ATPase is dispensable for the toxicity of VepA in yeast.

**Figure 3:**
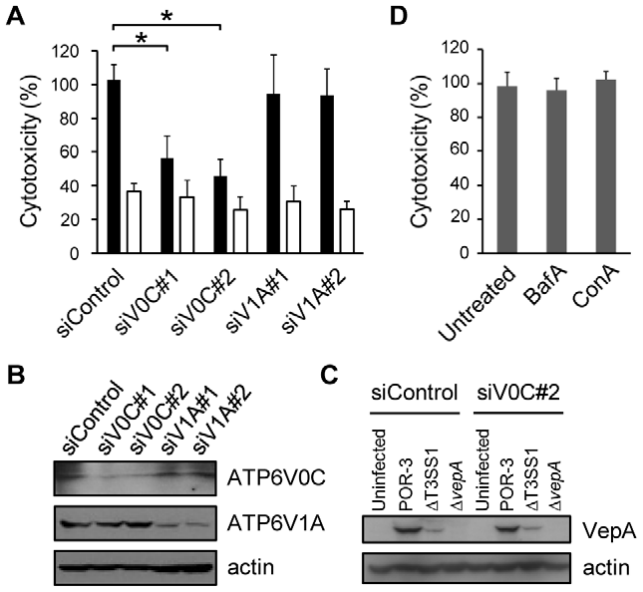
Subunit c of V-ATPase is involved in the cytotoxicity of VepA in HeLa cells. (A) HeLa cells treated with siRNAs were infected with the POR-3 (black bars) or Δ*vepA* strain (white bars) for 4 h, and cytotoxicity was measured using the LDH release assay. Data represent the mean ± SD. *P<0.01. (B) The knockdown level of the expression of the indicated proteins was confirmed by immunoblotting. (C) VepA translocation. HeLa cells treated with siRNAs were infected with the indicated strain for 3 h, and cell lysates were analyzed by immunoblotting with an anti-VepA antibody. An immunoblot for actin is shown as a loading control. (D) HeLa cells were left untreated or pre-treated with bafilomycin A (BafA) or concanamycin A (ConA) for 1 h and subsequently infected with POR-3 for 4 h. Cytotoxicity was determined using the LDH assay.

Next, we investigated the involvement of ATP6V0C, the human ortholog of yeast Vma3p, in VepA-mediated cytotoxicity. HeLa cells were transfected with siRNAs to knockdown ATP6V0C or ATP6V1A, subunit A of human V-ATPase. The transfected cells were infected with *V. parahaemolyticus* strain POR-3 [14], and the level of cytotoxicity was evaluated. The knockdown of ATP6V0C reduced infection-mediated cytotoxicity, but the knockdown of ATP6V1A did not (Figure 3A). The knockdown of expression of each protein was confirmed by immunoblotting (Figure 3B). We also determined whether the knockdown of ATP6V0C affected the translocation of VepA in cells. HeLa cells treated with siRNAs were infected with POR-3, ΔT3SS1 or Δ*vepA* strain for 3 h. The cell lysates were then analyzed by immunoblotting. The amount of VepA that was associated with the cells was not different between the control siRNA-treated cells and the ATP6V0C siRNA-treated cells (Figure 3C), indicating that the knockdown of ATP6V0C did not affect the translocation of VepA into cells. Moreover, pre-treatment with the pharmacological V-ATPase inhibitors, bafilomycin A and concanamycin A [22] at 100 nM, a concentration that is sufficient to prevent vesicular acidification as validated by LysoTracker staining (data not shown), did not prevent infection-mediated cytotoxicity (Figure 3D). These results indicate that ATP6V0C is involved in VepA-mediated cytotoxicity and that the function of the V-ATPase is not required for cytotoxicity, which is consistent with the yeast study described above (Figure 2E).

### VepA interacts with ATP6V0C

Next, we characterized the localization of VepA in *V. parahaemolyticus*-infected HeLa cells by biochemical subcellular fractionation. HeLa cells were infected with *V. parahaemolyticus* strains for 3 h to avoid the severe cytotoxicity that occurred at 4 h and fractionated into cytosolic, membrane/organelle, nuclear and cytoskeleton fractions. Each of the fractions was then analyzed by immunoblotting with an anti-VepA antibody. VepA was localized in the membrane/organelle fractions of cells infected with POR-3 or POR-3Δ*vepA*/*vepA* (Figure S3A). Furthermore, we fractionated the infected cells by ultracentrifugation (Figure S3B). VepA was mainly detected in the upper fractions, predominantly fraction 1, of cells infected with POR-3 or POR-3Δ*vepA*/*vepA*. This distribution of VepA is similar to that of V-ATPase (ATP6V1A and ATP6V0D1), which suggests that VepA may be associated with V-ATPases in infected cells.

**Figure 4:**
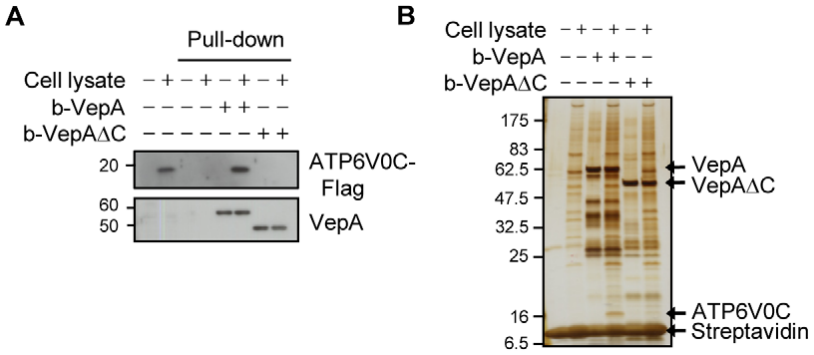
VepA is associated with ATP6V0C. (A) Pull-down assays with biotinylated VepA (b-VepA) or b-VepAΔC from lysates of 293T cells expressing ATP6V0C-Flag. Bound proteins were subjected to immunoblot analysis using an anti-Flag antibody. (B) Endogenous proteins of RAW264.7 cells that were pulled down with b-VepA or b-VepAΔC were separated by SDS-PAGE and detected by silver staining. Arrows indicate the locations of VepA, VepAΔC, streptavidin and ATP6V0C.

We next investigated whether VepA interacts with ATP6V0C. Biotinylated VepA (b-VepA) (Figure S4) was adsorbed onto streptavidin beads and incubated with lysates of 293T cells expressing ATP6V0C-Flag. Immunoblot analysis showed that ATP6V0C-Flag co-precipitated with b-VepA-immobilized beads (Figure 4A), indicating that VepA interacts with ATP6V0C. To further search for endogenous proteins that associate with VepA, proteins bound to VepA were pulled down from lysates of RAW264.7 cells, which are susceptible to T3SS1-induced cytotoxicity, and thus, have a potential for high expression of target molecules for VepA [10], and visualized by silver staining of SDS-PAGE gels (Figure 4B). We found a protein with a molecular weight of approximately 16 kDa that specifically associated with VepA. This protein band was excised, analyzed by liquid chromatography-tandem mass spectrometry (LC-MS/MS) and identified as ATP6V0C, as expected. By contrast, VepAΔC did not interact with Flag-tagged or endogenous ATP6V0C (Figure 4A and 4B). In addition, in cells infected with POR-3Δ*vepA*/*vepA*ΔC, the subcellular distribution of VepAΔC was different from that of VepA, and the protein was not enriched in fraction 1, which contains lysosomes (Figure S3B). ATP6V0C is an integral membrane protein that consists of 155 amino acids and is predicted to possess four transmembrane domains and two cytoplasmic loops [24] (Figure S5A). VepA is injected into the cytoplasm of host cells by the T3SS and therefore might target the cytoplasmic loops of ATP6V0C. To test this possibility, 293T cells expressing the cytoplasmic loops (Vc1: ATP6V0C 31–80 or Vc2: ATP6V0C 111–155) were infected with *V. parahaemolyticus*. Interestingly, the expression of Vc1 significantly reduced infection-mediated cytotoxicity (Figure S5B). In addition, Vc1 interacted with VepA in pull-down assays (Figure S5C). Taken together, our data indicate that ATP6V0C is a primary cellular target for VepA.

### Infection of *V. parahaemolyticus* induces the lysosomal rupture of host cells

**Figure 5:**
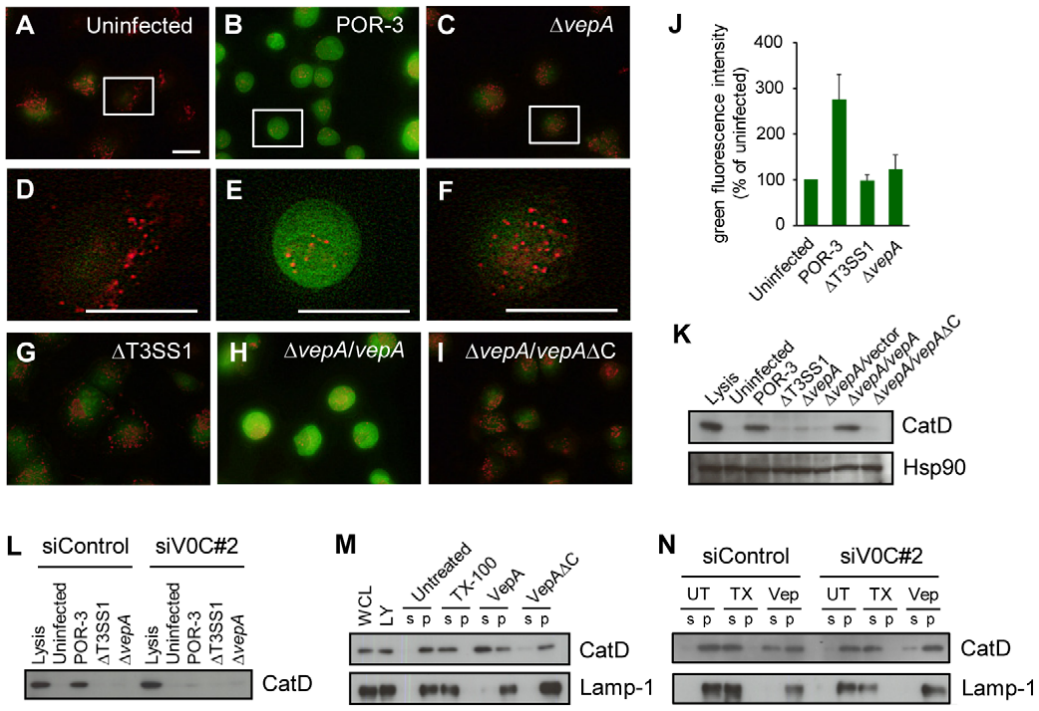
VepA induces lysosomal rupture. (A–I) AO relocation assays. HeLa cells were exposed to AO and then infected with the indicated strains for 3 h. AO fluoresces red in lysosomes and green in the cytosol. (D, E, F) High magnification of the area marked by white boxes in A, B and C. Scale bar, 20 µm. (J) Quantification of the green fluorescence intensity from the experiments shown in Figure 5A–G. (K) Cytosolic extracts of HeLa cells infected with the indicated strains for 3 h were subjected to immunoblot analysis using an anti-CatD antibody. Immunoblots for Hsp90 were performed as loading controls. Lysis represents the total release of CatD. (L) HeLa cells that were treated with siControl or siV0C#2 were infected with the indicated strains for 3 h, and cytosolic extracts were subjected to immunoblot analysis using an anti-CatD antibody. (M) Cell-free lysosome preparations were treated with VepA or VepAΔC for 2 h, centrifuged to pellet the lysosomes (p) and separate the supernatant (s) and subjected to immunoblot analysis for CatD. Immunoblots for Lamp-1, a lysosomal integral membrane protein, were performed as loading controls. Treating lysosomes with 1% Triton X-100 (TX-100) results in total lysis and was used as a positive control. Whole cell lysates (WCL) and lysosome preparations (LY) are also shown. (N) The effect of ATP6V0C knockdown on VepA-induced lysosomal rupture in a cell-free system. Lysosomes prepared from HeLa cells treated with siControl or siV0C#2, were left untreated (UT) or were treated with TX-100 or VepA. After pelleting the lysosomes (p) and separating the supernatant (s), both fractions were subjected to immunoblot analysis for CatD or Lamp-1.

V-ATPases are expressed on the membranes of various intracellular organelles, where they transport H^+^ across the membrane to generate and maintain acidic compartments. The lysosome is a major acidic compartment that contains various hydrolytic enzymes and functions as a digestive apparatus. The leakage of degradative contents from lysosomes into the cytosol is known to induce cell death, and the type of cell death that occurs is thought to be dependent on the extent of lysosomal damage, i.e., partial and moderate ruptures cause apoptosis, whereas more severe damage leads to necrosis [25], [26]. To investigate the integrity of lysosomes in infected cells, we used acridine orange (AO), which is a fluorochrome stain used for vital staining of lysosomes that exhibits red fluorescence when concentrated in lysosomes and green fluorescence when diffused in the cytosol [27]. AO-loaded HeLa cells were infected with *V. parahaemolyticus*. Enhanced green fluorescence was observed in cells infected with POR-3 or Δ*vepA*/*vepA* but not in cells infected with POR-3Δ*vepA* or ΔT3SS1, both of which are deficient in VepA (Figure 5A–I). Thus, the relocation of AO to the cytosol was VepA dependent.

To examine the extent of lysosomal leakage, we determined whether cathepsin D (CatD), a lysosomal aspartyl protease, translocates from lysosomes to the cytosol. CatD was detected in the cytosolic fractions of cells infected with POR-3 or POR-3Δ*vepA*/*vepA* but not in cells infected with VepA-deficient strains or POR-3Δ*vepA*/*vepA*ΔC, indicating that CatD was also released into the cytosol during infection in a VepA-dependent manner (Figure 5K). Furthermore, the knockdown of ATP6V0C partially reduced the release of CatD into the cytosol during infection (Figure 5L). We also treated AO-loaded HeLa cells with TDH, a *Vibrio* exotoxin. Although TDH does not contribute to the cytotoxicity of *V. parahaemolyticus* during infection, TDH attacks plasma membranes and functions as a pore-forming toxin that leads to cell death when HeLa cells are exposed to a high concentration of purified TDH [10], [28]. In contrast to infection with *V. parahaemolyticus*, treatment with TDH did not induce the relocation of AO to the cytosol (Figure S6A–D). Taken together, these results suggest that VepA-induced lysosomal rupture precedes plasma membrane disruption. In addition, pre-treatment of cells with U18666A or deferoxamine (DFO), both of which increase lysosomal membrane stability [29], [30], reduced infection-mediated cytotoxicity (Figure S6E and S6F).

### VepA ruptures lysosomes in a cell-free system

We next investigated the involvement of VepA in infection-mediated lysosomal rupture. Because VepA interacts with V-ATPase subunit c, which is highly expressed on lysosomal membranes, it is possible that VepA directly affects the integrity of lysosomes. We therefore examined whether VepA could directly rupture lysosomes in a cell-free system. Isolated lysosomes were incubated with VepA or VepAΔC. After precipitating the lysosomes, the release of CatD from the lysosomes into the supernatant was examined. For lysosomes treated with VepA, detectable levels of CatD were released into the supernatant. By contrast, the release of CatD was not observed in the supernatants from lysosomes treated with VepAΔC (Figure 5M). Therefore, these results suggest that the cytotoxic T3SS effector VepA alone is able to induce lysosomal rupture and the leakage of contents from lysosomes. Finally, we examined the effect of knockdown of ATP6V0C on VepA-induced lysosomal rupture in the cell-free system. Lysosomes that were isolated from control siRNA- or ATP6V0C siRNA-treated cells, were treated with VepA. For lysosomes from ATP6V0C-silenced cells, CatD release was partially decreased (Figure 5N), indicating that the knockdown of ATP6V0C reduces VepA-induced lysosomal rupture. Thus, these results indicate that ATP6V0C is involved in VepA-induced lysosomal rupture.

## Discussion

Pathogens manipulate host cell death to facilitate their ability to cause infections [31], [32]. The bacterial pathogen *V. parahaemolyticus* elicits T3SS1-dependent non-apoptotic and caspase-independent cell death during infection (Figure S1) [11], [12]. Among the effectors translocated by the T3SS1 of *V. parahaemolyticus*, VepA is thought to play an important role in *V. parahaemolyticus*-induced cytotoxicity because the Δ*vepA* strain has lost the majority of the cytotoxicity of the wild-type [14], [15]. In this study, we showed that VepA itself is cytotoxic and acts only inside cells, not outside cells (Figure 1). It is reasonable for VepA to be cytotoxic only inside cells because it is injected into the cytoplasm of host cells by T3SS. Among the vibrios, *V. alginolyticus*, *V. harveyi* and *V. tubiashii* possess T3SSs that are highly homologous to *V. parahaemolyticus* T3SS1 [8]. The *V. alginolyticus* T3SS exhibits cytotoxicity in mammalian and fish cells [33], [34]. Because VepA is also conserved among these species, it is possible that VepA homologs are involved in the cytotoxicity of other *Vibrio* species.

Our yeast genome-wide screen revealed that subunit c of V-ATPase is indispensable for the toxicity of VepA in yeast (Figure 2). In HeLa cells, knockdown of ATP6V0C with siRNA decreased VepA-mediated cytotoxicity significantly but not completely (Figure 3), presumably due to the insufficient knockdown efficiency of ATP6V0C, as validated in Figure 3B. The expression of GFP-Vc1, a cytoplasmic loop of ATP6V0C, also reduced the cytotoxicity significantly but only partially (Figure S5B). GFP-fused cytoplasmic loops exhibited a diffuse cytoplasmic pattern and no lysosomal localization (data not shown), which may result in insufficient competition with ATP6V0C. Our pull-down assays suggested that VepA prefers Vc1, but Vc2 was also weakly associated with VepA (Figure S5C). An alternative explanation for the residual cytotoxicity is that VepA may recognize not only the cytoplasmic loop 1 of ATP6V0C but also the more complicated structure of ATP6V0C. Thus, although we cannot exclude the possibility that VepA has other unknown cellular targets that may also be involved in cytotoxicity at this stage, VepA-mediated cytotoxicity in HeLa cells at least partially requires subunit c of V-ATPase, supporting the validity of our yeast genome-wide screen.

V-ATPase is composed of multisubunits, all of which are required for the proton transport activity. The V-ATPase complex is divided into the two domains, V_1_ and V_0_ domains, which can assemble independently [22]. Subunit c of V-ATPase, a component of the V_0_ domain, is also known as ductin, which is thought to form a channel by itself [24]. Therefore, even in the absence of other ATPase components, it is possible that molecular complexes containing subunit c of V-ATPase are expressed in the membrane, although these complexes may not function as ATPases. In yeast, in the assembly of the V_0_ domain, deletions in the V_0_ subunits have been reported to result in the failure of the V_0_ domain to localize to vacuolar membranes [35], [36]. However, in our yeast toxicity assays, presented in Figure 2, VepA was shown to be toxic to the V_0_ subunit mutants except for Δ*vma3*. Therefore, although it should be noted that VepA was ectopically overexpressed in yeast in these experiments, we cannot completely exclude the possibility that VepA may target subunit c of V-ATPase, which is also located in compartments other than vacuolar membranes. This possibility will be explored in the future.

Biochemical cell fractionation revealed the distribution of VepA is almost identical to that of V-ATPases (Figure S3). V-ATPases are expressed in acidic organelles and at plasma membranes [22]. Indeed, the distribution of V-ATPases was similar to that of lysosomes and plasma membranes in Figure S3B. By contrast, the distribution of VepAΔC, which did not interact with ATP6V0C, was different from that of wild-type VepA, suggesting that VepA is associated with these organelles via the interaction with subunit c of V-ATPases. V-ATPases are evolutionally conserved among eukaryotic cells [23]. A bacterial virulence factor that targets such a broadly distributed molecule among species is an efficient way to ensure a wide range of host species susceptibility. The *Pseudomonas aeruginosa* pigment pyocyanin and *Legionella pneumophila* SidK inactivate V-ATPase [37], [38]. Pyocyanin has been implicated in chronic *P. aeruginosa* infection in cystic fibrosis [39]. However, some yeast V-ATPase mutant strains are more sensitive to pyocyanin than the wild-type strain, a property that is distinct from the toxicity of VepA, which is not toxic to Δ*vma3*. SidK, a type IV secreted effector, targets subunit A of V-ATPase, which contains the ATP hydrolytic site, and inhibits ATP hydrolysis to prevent the proton pump function of V-ATPase and phagosomal acidification [38]. By contrast, our data indicate that, although VepA targeted subunit c of V-ATPase, the function of V-ATPase is dispensable for the toxicity of VepA (Figure 2 and Figure 3). Unlike VepA, which showed severe and acute cytotoxic activity within 4 h after infection (Figure 1 and S6C), SidK appeared not to be cytotoxic during the early infection period because macrophages loaded with the SidK protein survive longer than 24 h [38]. This difference may reflect the difference between the infection strategies of the two pathogens: *V. parahaemolyticus* is an extracellular pathogen that causes acute infection [5], whereas *L. pneumophila* is an intracellular pathogen that survives and replicates in phagosomes and therefore needs to avoid phagosomal acidification [40].

Infection with *V. parahaemolyticus* caused VepA injection-dependent leakage of the lysosomal contents (Figure 5). Lysosomes are described as “suicide bags”, because they contain numerous hydrolases [41]. Lysosomotropic agents such as H_2_O_2_ and sphingosine induce lysosomal membrane permeabilization and subsequent cell death [42]. Lysosomal rupture-induced cell death is thought to be dependent on the extent of lysosomal damage; partial and moderate rupture causes apoptosis, whereas more severe damage leads to necrosis [25], [26]. *V. parahaemolyticus* infection-mediated leakage of lysosomal contents was observed not only with a small-molecule dye AO but also with the protein CatD, suggesting that extensive lysosomal membrane permeabilization occurs within hours of infection. Notably, our cell-free assay showed that VepA is sufficient to reproduce infection-mediated lysosomal rupture, whereas VepAΔC, which is deficient for the association with ATP6V0C, could not induce the leakage of lysosomal contents. Moreover, the knockdown of ATP6V0C reduced VepA-induced lysosomal rupture (Figure 5L and 5N), indicating that ATP6V0C is required of VepA-induced lysosomal rupture. Thus, our data provide the first example of a bacterial T3SS effector that ruptures lysosomes directly to induce cell death.

Various bacterial effectors are known to have enzymatic activities such as proteolytic processing and post-translational modification [7]. However, despite experimental efforts, we could not detect the processing or modification of ATP6V0C by VepA in this study. Although it is not yet known how the interaction of VepA with ATP6V0C leads to destabilization of lysosomal membranes, ATP6V0C may serve as a scaffold for VepA, facilitating the access of VepA to lysosomal membranes and subsequent lysosomal rupture. Alternatively, the association of VepA with ATP6V0C may destabilize the ATP6V0C complex, which may result in lysosomal destabilization. Two lysosome stabilizers, U18666A and DFO, partially inhibited T3SS1-dependent cytotoxicity (Figure S6E and S6F). U18666A inhibits transport of cholesterol from lysosomes to the ER, which causes the accumulation of cholesterol in lysosomes [29]. DFO is known to protect against H_2_O_2_-induced lysosome rupture by chelating intralysosomal iron [30]. In contrast to those direct effects on lysosomal stabilization, the incubation of heat shock protein 70 (Hsp70) with cells, which is reported to stabilize lysosomes by an indirect effect that stimulates acid sphingomyelinase activity [43], failed to prevent T3SS1-dependent cytotoxicity (data not shown). Thus, these results suggest that VepA physically destabilizes lysosomal membranes.

The cell death induced by *V. parahaemolyticus* has also been reported to be associated with autophagy [11]. Although autophagy is well known to promote cell survival in response to various stimuli [44], recent studies have indicated that autophagy also plays a role as an executor of cell death in some aspects [45], [46]. However, the role of autophagy induced by *V. parahaemolyticus* in cell death is unclear. A previous report showed that *V. parahaemolyticus* does not activate autophagy in *Atg5*-/- murine embryonic fibroblasts, indicating that *V. parahaemolyticus*-induced autophagy is dependent on ATG5 [16]. The results of this study were consistent with those of a prior study, demonstrating that ATG5 depletion by siRNA inhibited *V. parahaemolyticus*-induced autophagy in HeLa cells (Figure S1E). However, ATG5 depletion did not affect T3SS1-dependent cytotoxicity (Figure S1C). Moreover, Δ*atg5* and Δ*atg8* yeast strains, both of which are deficient in the autophagic process, were not resistant to VepA (Figure S2B). Indeed, although there is a link between autophagy and lysosomal biogenesis [47], our results indicate that autophagy does not contribute to the cell death induced by *V. parahaemolyticus*. Thus, *V. parahaemolyticus* induces “cell death with autophagy” but not “cell death by autophagy” [48]. It is thus possible that autophagy may play a protective role against cytotoxicity of *V. parahaemolyticus*. It has also been reported that VepA is linked to the activation of MAPK signaling when cells are infected with *V. parahaemolyticus* [19]. MAPK cascades are activated by various stimuli including cellular stress [49]. A recent report has shown that deficiency in the tumor susceptibility gene 101 leads to lysosomal distention as well as induction of autophagy and MAPK activation [50]. Thus, it is possible that lysosomal stress may be linked to induction of stress response, such as activation of MAPK and induction of autophagy.

In conclusion, we demonstrated that VepA targets ATP6V0C and ruptures lysosomes. Although the mechanism of lysosomal rupture by VepA warrants further exploration, it is intriguing that a pathogenic bacterium induces cell death by causing the host cell to leak its own dangerous content, which is akin to striking the Achilles' heel of the host cell.

## Materials and Methods

### Bacterial and yeast strains

The *Vibrio parahaemolyticus* strains POR-3 (RIMD2210633Δ*tdhAS*Δ*vcrD2*, which is T3SS2 deficient) and POR-3Δ*vcrD1* (which lacks both T3SS1 and T3SS2) were described previously [9], [14]. The POR-3Δ*vepA* strain was created as described previously [14]. To complement the mutant with *vepA* or *vepA*ΔC (1–400), the pSA-19CS-MCS vector was used as described previously [14]. *Saccharomyces cerevisiae* BY4730 (*MATa leu2*Δ*0 met15*Δ*0 ura3*Δ*0*) was obtained from Open Biosystems. The plasmids and oligonucleotide primers used in this study are listed in Table S1.

### Reagents and antibodies

Bafilomycin A and concanamycin A (both used at 100 nM) were purchased from Sigma. Acridine orange was purchased from Invitrogen. U18666A, deferoxamine, SP600125, U0126 and SB20358 were purchased from Sigma. The pan-caspase inhibitor Z-VAD-FMK was from Medical & Biological Laboratories. The following antibodies were used: antibodies against Lamp-1, CD49b, Grb78, GM130, nucleoporin-62, Bcl-2 and Hsp90 (BD Biosciences); antibodies against β-actin, Flag and poly-histidine (Sigma); anti-ATP6V1A (Abnova); anti-ATP6V0D1 (Abcam); anti-CatD (Cell Signaling Technology); HRP-conjugated streptavidin (Pierce); anti-ATP6V0C (Millipore); HRP-conjugated anti-GFP (Miltenyi Biotech); and anti-ATG5 and anti-LC3 (Medical & Biological Laboratories). The anti-VepA antibody has been described elsewhere [15].

### Cell culture and cytotoxicity assay

HeLa, RAW264.7 and 293T cells were maintained in DMEM (Sigma) containing 10% FBS (Sigma) at 37°C in 5% CO_2_. For infections, bacteria were used to challenge HeLa cells at a multiplicity of infection of 10 [10]. The cytotoxicity assay was performed using the CytoTox96 Non-Radioactive Cytotoxicity Assay Kit (Promega) as previously described [9].

### Yeast toxicity assay

Yeast was transformed using the Frozen-EX Transformation Kit II (Zymo Research). Transformants were grown on SC plates lacking uracil and containing 2% glucose (SC-Ura+Glc) at 30°C for 48–72 h. Growing colonies were picked and cultured in SD media lacking uracil and containing 2% glucose (SD-Ura+Glc). Yeast was washed once with 0.67% yeast nitrogen broth without amino acids and adjusted to an optical density of 1 at 600 nm. SC plates lacking uracil and containing glucose or galactose (SC-Ura+Gal) were spotted with 5-µl aliquots of 10-fold serial dilutions and then incubated at 30°C for 72 h. For the complementation of *VMA3* in Δ*vma3*, a *LEU2* plasmid pRS415, encoding *VMA3* along with 500 bp upstream and downstream of *VMA3* was used.

### Protein expression

His-tagged VepA or VepAΔC protein was purified as described previously [15]. To construct pATP6V0C-Flag, cDNA for ATP6V0C with a Flag-tag and a stop codon at the C-terminus was inserted into pEGFP-N1, yielding the ATP6V0C-Flag construct without GFP.

### DNA transfection

For DNA transfection, 293T cells were used. To transfect the VepA constructs, 293T cells were seeded on collagen-coated coverslips in 6-well plates and grown for 24 h. The cells were then transfected with pEGFP-C1, pEGFP-VepA or pEGFP-VepAΔC using Lipofectamine 2000 (Invitrogen). The cells were fixed with 3% paraformaldehyde and permeabilized with 0.2% Triton X-100 (TX-100) 24 h post-transfection and then stained with rhodamine-phalloidin. The coverslips were analyzed by fluorescence microscopy using a Biozero BZ-8100 microscope (Keyence). To infect cells expressing the ATP6V0C constructs, 293T cells seeded in 96-well plates were transfected with pEGFP-C1, pEGFP-Vc1 or pEGFP-Vc2 using Lipofectamine LTX (Invitrogen). After 24 h, the cells were infected with POR-3 at a multiplicity of infection of 10 for 3 h and then assessed using the cytotoxicity assay.

### Protein transfection

Proteins were introduced into cells using GenomONE-Neo (Ishihara Sangyo) according to the manufacturer's instructions. Briefly, 10 µl of HVJ-envelope was mixed with 2.5 µl of Reagent A and incubated for 5 min on ice. The suspension was mixed with 5 µg of protein and then with 1.5 µl of Reagent B. After centrifugation at 10,000 *g* for 5 min, the supernatant was removed. The pellets were resuspended in 15 µl of Reagent Buffer, followed by the addition of 2.5 µl of Reagent C to complete the preparation of the HVJ-liposome-including proteins. A one-eighth aliquot of the envelope was added to cells in a 96-well plate. The cells were incubated at 37°C for 1.5 h and then washed with phosphate-buffered saline (PBS). The medium was replaced with fresh medium, and the cells were incubated at 37°C for 2.5 h, and cytotoxicity was determined by assaying the cellular dehydrogenase activity using Cell Counting Kit-8 (Dojindo).

### Screening for VepA-resistant strains

Yeast MATa haploid non-essential gene knockout strains from Open Biosystems [21] were grown in YPD medium in 96-well plates at 30°C. All of the strains from a single plate were then pooled into a single culture, and transformed with p426-VepA as described above. Transformants were plated on SC-Ura+Gal and incubated at 30°C for 3 days. To determine the plating efficiency, transformants were also plated on SC-Ura+Glc. Colonies grown on galactose plates were picked and incubated in SD-Ura+Glc. Chromosomal DNA was purified by phenol/chloroform extraction with glass beads as described elsewhere [51]. Barcode-tag sequence regions of the extracted yeast DNA were amplified by polymerase chain reaction. Then, amplicons were purified using ExoSAP-IT (GE healthcare) and sequenced to verify the barcode-tag. We eliminated mutants that were only recovered in a single clone to exclude false positives. For the second selection, the identified mutants were individually transformed with p426-VepA, and their growth on SC-Ura+Gal was examined. A flowchart summarizing the protocol for the yeast genome-wide screen used in this study is shown in Figure 2B.

### siRNA knock-down

Ambion's Silencer Select Validated siRNAs and Silencer Select Pre-designed siRNAs were used to knockdown ATP6V0C and ATP6V1A, respectively. HeLa cells were reverse-transfected with 20 nM Silencer Negative Control#2 siRNA or with two independent siRNAs targeting ATP6V0C (V0C#1; s80, V0C#2; s81) or ATP6V1A (V1A#1; s1791, V1A#2; s1792) using the siPORT *NeoFX* Transfection Agent (Ambion). Then, 96 h after siRNA transfection, the cells were infected with *V. parahaemolyticus* strain POR-3 as described above. To determine the knockdown efficiency, immunoblot analysis was performed using anti-ATP6V1A and anti-ATP6V0C antibodies. To deplete ATG5, FlexiTube siRNAs (Qiagen) were used. HeLa cells were transfected with AllStars Negative Control siRNAs or two separate siRNAs targeting ATG5 (ATG5_2; #1, ATG5_6; #2) using HiPerFect Transfection Reagent (Qiagen). Then, 72 h post-transfection, the cells were infected with strain POR-3 for 4 h, and cytotoxicity was evaluated.

### Pull-down assay

For pull-down assays, 293T cells expressing ATP6V0C-Flag or RAW264.7 cells were lysed with RIPA buffer containing a protease inhibitor cocktail (Sigma). VepA and VepAΔC were biotinylated using EZ-Link Sulfo-NHS-SS-Biotinylation kits (Pierce). Biotinylated proteins were captured with Streptavidin Sepharose beads (GE healthcare). Then, VepA- or VepAΔC-immobilized beads were incubated with cell lysates on a rotary shaker for 2 h at 4°C. The beads were washed with RIPA buffer five times, and resuspended in SDS-loading buffer to elute bound proteins from the beads. Eluates were subjected to SDS-PAGE and immunoblot analysis or LC-MS/MS analysis as described previously [52]. Proteins were identified by a database search using Mascot (Matrix Science).

### Cell fractionation

Subcellular fractionation was performed using the ProteoExtract Subcellular Proteome Extraction Kit (Calbiochem) according to the manufacturer's instructions. For organelle fractionations, we used the Lysosome Enrichment Kit for Tissue and Cultured Cells (Pierce) according to the manufacturer's instructions. Briefly, the lysates from infected cells were applied to a 15–30% OptiPrep density gradient. After ultra-centrifugation at 150,000 *g* for 2 h at 4°C in a SW55 rotor (Beckman Coulter), 800-µl fractions were collected from the top of the tube. Each fraction was diluted with PBS and centrifuged at 20,000 *g* for 30 min at 4°C. After removing the supernatants, the pellets were dissolved with SDS-loading buffer and subjected to SDS-PAGE and immunoblot analysis. To prepare the cytosolic fraction, HeLa cells were solubilized with saponin buffer (0.1% saponin and protease inhibitors in PBS) for 15 min on ice. After centrifugation at 15,000 *g* for 10 min, the supernatants were collected. The cytosolic fraction was free of membranes as verified by immunoblots using an anti-Lamp-1 antibody. Lysis refers to treating the cells with 1% TX-100 to induce the total release of CatD as a positive control.

### AO relocation assay

HeLa cells grown on 35-mm glass bottom dishes were treated with 2 µg ml^−1^ AO at 37°C for 15 min. After washing with PBS, the medium was replaced with fresh medium, followed by infection or exposure to TDH. Fluorescence micrographs of AO-stained cells were obtained using the same fluorescence intensity and exposure time. The intensity of green fluorescence was quantitated with a BZ Analyzer II (Keyence).

### Cell-free assay

The cell-free lysosome-enriched fraction was prepared as described [53]. Briefly, HeLa cells were homogenized in ice-cold TS buffer (10 mM Tris-HCl, pH 7.5, 250 mM sucrose and protease inhibitors). Then, the cells were centrifuged at 1,000 *g* to pellet the nuclei and cell debris. The supernatants were centrifuged at 3,000 *g*. The supernatants were further centrifuged at 17,000 *g*, and the resulting pellets, which contained the lysosomes, were resuspended with PBS and used as a cell-free lysosomal preparation. Then, 50-µl aliquots of prepared lysosomes (50 µg) were exposed to 10 µg ml^−1^ VepA or VepAΔC for 2 h at 37°C. The reaction mixture was centrifuged at 20,000 *g* for 30 min at 4°C to separate the pellets, which contained the lysosomes, from the supernatants, which contained the material that leaked from the lysosomes. Both fractions were subjected to SDS-PAGE and immunoblot analyses using anti-CatD and anti-Lamp-1 antibodies.

### Monitoring autophagy

To induce autophagy, HeLa cells were starved in Earle's balanced salt solution for 6 h or infected with *V. parahaemolyticus* POR-3 or Δ*vepA* for 3 h. For the nutrient-rich condition, cells were cultured in DMEM containing 10% FBS. To measure autophagy, the conversion of LC3-I to LC3-II was monitored by immunoblotting as previously described [54].

### Statistical analysis

Statistical analysis was performed using the two-tailed Student's *t*-test.

## Supporting Information

Figure S1 **VepA-dependent cytotoxicity is independent of autophagy, caspases and MAPK activity.** (A) The effect of a pan-caspase inhibitor on cytotoxicity. HeLa cells were left untreated or were pre-treated for 1 h with Z-VAD-FMK (at 100 µM, a concentration that is sufficient to inhibit apoptosis induced by treatment with 1 µM staurosporine for 6 h; data not shown), followed by infection with the POR-3 strain for 4 h. Cytotoxicity was evaluated using the LDH release assay. (B) The effects of MAPK inhibitors on cytotoxicity. HeLa cells were left untreated or were pre-treated for 1 h with SP600125 (a JNK inhibitor, at 15 µM), U0126 (a MEK1/2 inhibitor, at 10 µM) or SB20358 (a p38 MAPK inhibitor, at 5 µM), and then infected with the POR-3 strain for 4 h. Cytotoxicity was evaluated using the LDH release assay. The concentration of each inhibitor was sufficient to inhibit MAPK signaling activated by 100 nM phorbol 12-myristate 13-acetate for 15 min or 25 µg/ml anisomycin for 30 min (data not shown). (C) HeLa cells were transfected with control siRNA or two independent siRNAs targeting ATG5. After 72 h, the cells were infected with POR-3 for 4 h, and cytotoxicity was evaluated using the LDH release assay. The values represent the mean ± SD for a minimum of three independent experiments. (D) Knockdown of ATG5 protein expression in siRNA-treated cells was validated by immunoblot analysis. An immunoblot for actin is also shown as a loading control. (E) HeLa cells treated with control siRNA (Co) or two ATG5 siRNAs (#1, #2) were left uninfected, were infected with POR-3 or Δ*vepA* for 3 h, or were starved for 6 h. The lysates were subjected to immunoblot analysis using anti-LC3 and anti-actin antibodies.
(TIF)

Figure S2 **Complementation of the** ***VMA3*** **gene in the Δ** ***vma3*** **strain restored the toxicity of VepA.** (A) The growth patterns of the 10-fold serial dilutions of the Δ*vma3* strains harboring p426-VepA and pRS415 or p426-VepA and pRS415 encoding *VMA3* on SC lacking leucine and uracil and containing Glc (SC-Leu-Ura+Glc) and SC-Leu-Ura containing Gal plates are shown. (B) The sensitivity to VepA of yeast autophagy mutants (Δ*atg5* and Δ*atg8*) and MAPK mutants (Δ*kss1*, Δ*hog1*, Δ*fus3*, Δ*slt2* and Δ*smk1*).
(TIF)

Figure S3 **Subcellular localization of** ***V. parahaemolyticus*** **-infected cells.** (A) HeLa cells were infected with the indicated strains for 3 h and fractionated as described in Materials and Methods. Subcellular fractions were subjected to immunoblot analysis using an anti-VepA antibody (left panel). In the right panel, the purity of these fractions was confirmed by immunoblot analysis for Hsp90 (cytosol), CD49b and Lamp-1 (membrane and organelle, respectively), and Nucleoporin p62 (nucleus). (B) Cellular organelle fractionation of HeLa cells infected with the indicated strains for 3 h. Each fraction were analyzed by immunoblotting to determine the locations of VepA, subunit A of the V_1_ domain of V-ATPase (ATP6V1A), subunit d of the V_0_ domain of V-ATPase (ATP6V0D), Lamp-1 (lysosomes), CD49b (plasma membranes), Grb78 (endoplasmic reticulum), Bcl-2 (mitochondria) and GM130 (Golgi apparatus).
(TIF)

Figure S4 **Biotinylated VepA retains cytotoxicity.** (A) The biotinylation of VepA or VepAΔC was confirmed by immunoblot analysis using HRP-conjugated streptavidin (SA). VepA and its derivatives, which all contain a histidine-tag, were detected using an anti-histidine-tag antibody. (B) Biotinylated VepA (b-VepA) was delivered into HeLa cells using the HVJ envelope, and after 4 h, cytotoxicity was determined using the Cell Counting Kit-8 (Dojindo). The values represent the mean ± SD for a minimum of three independent experiments.
(TIF)

Figure S5 **VepA prefers to bind cytoplasmic loop 1 of ATP6V0C.** (A) A schematic outline of the location of the truncated derivatives of ATP6V0C (155 amino acids) is shown (left). Vc1 (ATP6V0C 31–80) includes cytoplasmic loop 1, which is located between transmembrane domains (TMs) I and II, and Vc2 (ATP6V0C 111–155) includes loop 2, which is located between TMs III and IV. The disposition of ATP6V0C is also shown (right). (B) 293T cells transfected with pEGFP-C1, pEGFP-Vc1 or pEGFP-Vc2 were infected with POR-3 for 3 h. Cytotoxicity was determined using the LDH release assay. The values represent the mean ± SD of a minimum of three independent experiments. (C) Pull-down assays with b-VepA from lysates of 293T cells expressing GFP, GFP-Vc1 or GFP-Vc2. Bound proteins were eluted and subjected to immunoblot analysis using an anti-GFP antibody.
(TIF)

Figure S6 **Time-course analysis of HeLa cells infected with** ***V. parahaemolyticus*** **or treated with TDH.** (A, B) AO relocation assay in HeLa cells infected with *V. parahaemolyticus* POR-3 or treated with 100 µg ml^−1^ TDH toxin for the indicated times (min). Representative high magnifications of the areas indicated by white boxes in the top panels are shown in the bottom panels. Scale bar, 20 µm. (C, D) The cytotoxicity of HeLa cells infected with *V. parahaemolyticus* POR-3 (C) or treated with TDH (D) for the indicated times was evaluated by the LDH release assay. (E, F) The effects of lysosomal membrane stabilizers on the cytotoxicity induced by *V. parahaemolyticus*. HeLa cells were left untreated (UT) or were treated with U18666A (0.5 µg ml^−1^, 48 h) or deferoxamine (DFO; 10 µM, 24 h), and infected with POR-3 for 4 h. The cytotoxicity was determined using the LDH release assay. *P<0.01.
(TIF)

Table S1 **The plasmids and primers used in this study.**
(DOC)
