# Supplementary figures and images for: A Cytotoxic Type III Secretion Effector of Vibrio parahaemolyticus Targets Vacuolar H+-ATPase Subunit c and Ruptures Host Cell Lysosomes
Source: PLoS Pathog. 2012 Jul 19;8(7):e1002803. doi: 10.1371/journal.ppat.1002803 (PMC3400558; doi:10.1371/journal.ppat.1002803)

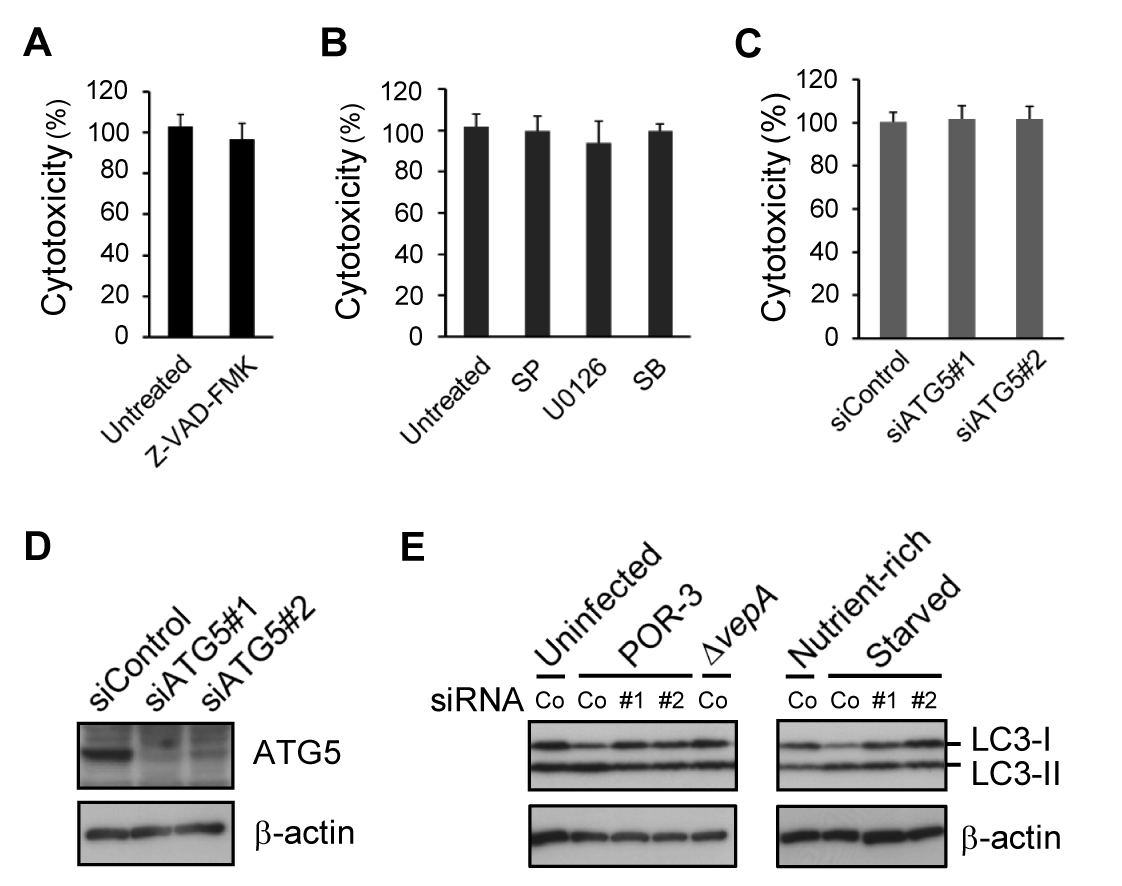

Supplement: Figure S1 — VepA-dependent cytotoxicity is independent of autophagy, caspases and MAPK activity. (A) The effect of a pan-caspase inhibitor on cytotoxicity. HeLa cells were left untreated or were pre-treated for 1 h with Z-VAD-FMK (at 100 µM, a concentration that is sufficient to inhibit apoptosis induced by treatment with 1 µM staurosporine for 6 h; data not shown), followed by infection with the POR-3 strain for 4 h. Cytotoxicity was evaluated using the LDH release assay. (B) The effects of MAPK inhibitors on cytotoxicity. HeLa cells were left untreated or were pre-treated for 1 h with SP600125 (a JNK inhibitor, at 15 µM), U0126 (a MEK1/2 inhibitor, at 10 µM) or SB20358 (a p38 MAPK inhibitor, at 5 µM), and then infected with the POR-3 strain for 4 h. Cytotoxicity was evaluated using the LDH release assay. The concentration of each inhibitor was sufficient to inhibit MAPK signaling activated by 100 nM phorbol 12-myristate 13-acetate for 15 min or 25 µg/ml anisomycin for 30 min (data not shown). (C) HeLa cells were transfected with control siRNA or two independent siRNAs targeting ATG5. After 72 h, the cells were infected with POR-3 for 4 h, and cytotoxicity was evaluated using the LDH release assay. The values represent the mean ± SD for a minimum of three independent experiments. (D) Knockdown of ATG5 protein expression in siRNA-treated cells was validated by immunoblot analysis. An immunoblot for actin is also shown as a loading control. (E) HeLa cells treated with control siRNA (Co) or two ATG5 siRNAs (#1, #2) were left uninfected, were infected with POR-3 or ΔvepA for 3 h, or were starved for 6 h. The lysates were subjected to immunoblot analysis using anti-LC3 and anti-actin antibodies. (TIF) [file ppat.1002803.s001.tif]

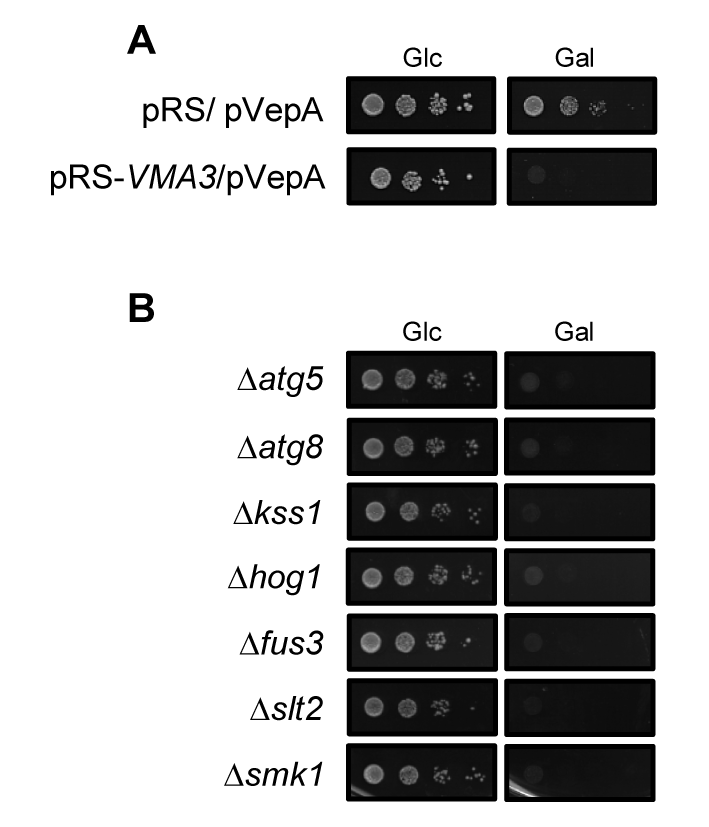

Supplement: Figure S2 — Complementation of the VMA3 gene in the Δ vma3 strain restored the toxicity of VepA. (A) The growth patterns of the 10-fold serial dilutions of the Δvma3 strains harboring p426-VepA and pRS415 or p426-VepA and pRS415 encoding VMA3 on SC lacking leucine and uracil and containing Glc (SC-Leu-Ura+Glc) and SC-Leu-Ura containing Gal plates are shown. (B) The sensitivity to VepA of yeast autophagy mutants (Δatg5 and Δatg8) and MAPK mutants (Δkss1, Δhog1, Δfus3, Δslt2 and Δsmk1). (TIF) [file ppat.1002803.s002.tif]

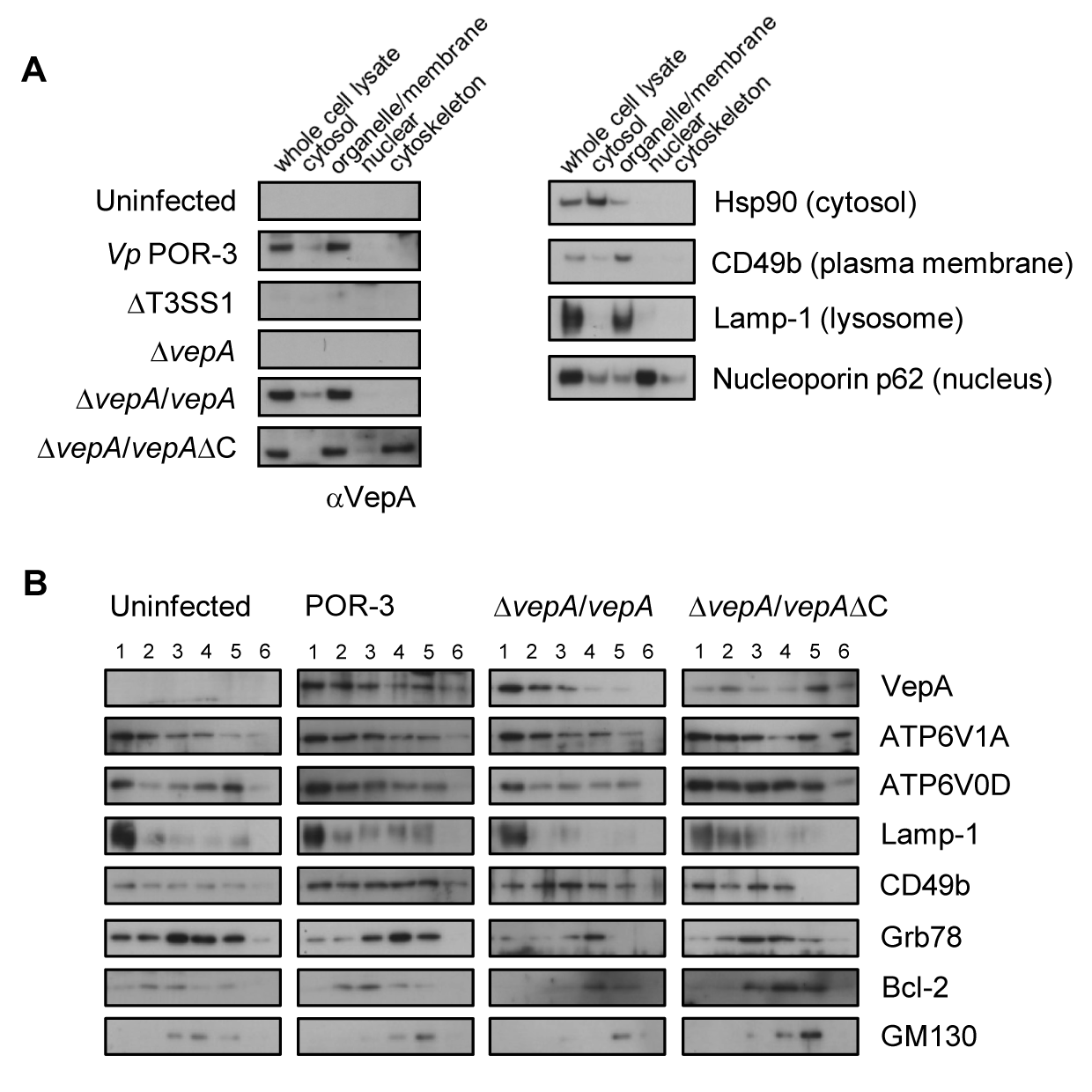

Supplement: Figure S3 — Subcellular localization of V. parahaemolyticus -infected cells. (A) HeLa cells were infected with the indicated strains for 3 h and fractionated as described in Materials and Methods. Subcellular fractions were subjected to immunoblot analysis using an anti-VepA antibody (left panel). In the right panel, the purity of these fractions was confirmed by immunoblot analysis for Hsp90 (cytosol), CD49b and Lamp-1 (membrane and organelle, respectively), and Nucleoporin p62 (nucleus). (B) Cellular organelle fractionation of HeLa cells infected with the indicated strains for 3 h. Each fraction were analyzed by immunoblotting to determine the locations of VepA, subunit A of the V1 domain of V-ATPase (ATP6V1A), subunit d of the V0 domain of V-ATPase (ATP6V0D), Lamp-1 (lysosomes), CD49b (plasma membranes), Grb78 (endoplasmic reticulum), Bcl-2 (mitochondria) and GM130 (Golgi apparatus). (TIF) [file ppat.1002803.s003.tif]

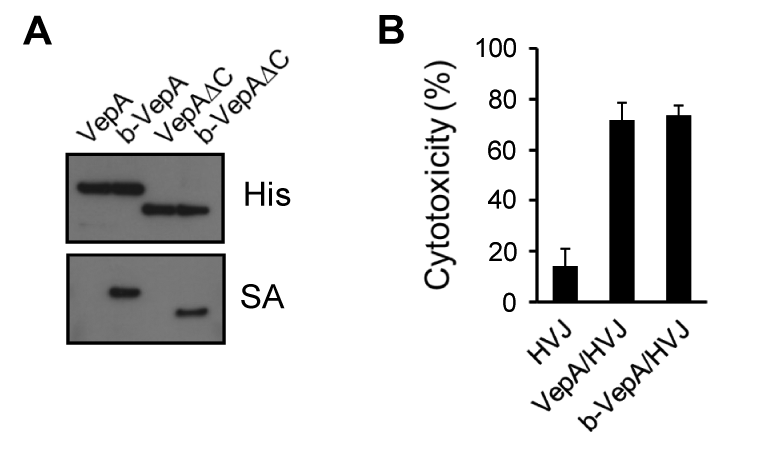

Supplement: Figure S4 — Biotinylated VepA retains cytotoxicity. (A) The biotinylation of VepA or VepAΔC was confirmed by immunoblot analysis using HRP-conjugated streptavidin (SA). VepA and its derivatives, which all contain a histidine-tag, were detected using an anti-histidine-tag antibody. (B) Biotinylated VepA (b-VepA) was delivered into HeLa cells using the HVJ envelope, and after 4 h, cytotoxicity was determined using the Cell Counting Kit-8 (Dojindo). The values represent the mean ± SD for a minimum of three independent experiments. (TIF) [file ppat.1002803.s004.tif]

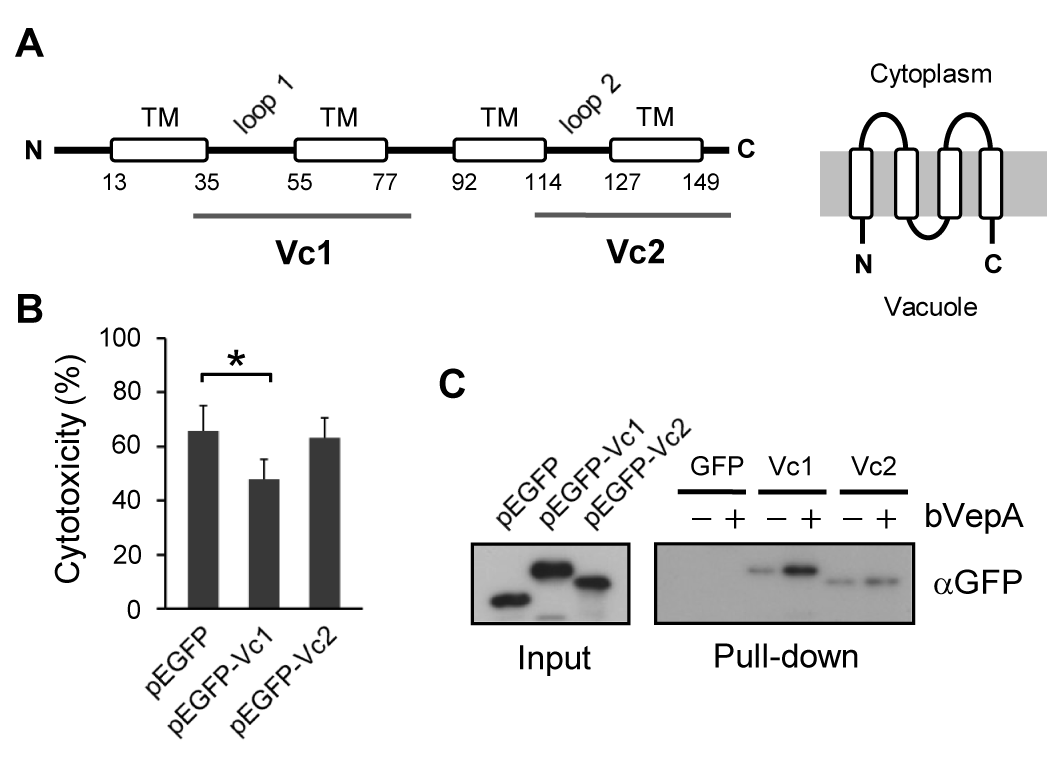

Supplement: Figure S5 — VepA prefers to bind cytoplasmic loop 1 of ATP6V0C. (A) A schematic outline of the location of the truncated derivatives of ATP6V0C (155 amino acids) is shown (left). Vc1 (ATP6V0C 31–80) includes cytoplasmic loop 1, which is located between transmembrane domains (TMs) I and II, and Vc2 (ATP6V0C 111–155) includes loop 2, which is located between TMs III and IV. The disposition of ATP6V0C is also shown (right). (B) 293T cells transfected with pEGFP-C1, pEGFP-Vc1 or pEGFP-Vc2 were infected with POR-3 for 3 h. Cytotoxicity was determined using the LDH release assay. The values represent the mean ± SD of a minimum of three independent experiments. (C) Pull-down assays with b-VepA from lysates of 293T cells expressing GFP, GFP-Vc1 or GFP-Vc2. Bound proteins were eluted and subjected to immunoblot analysis using an anti-GFP antibody. (TIF) [file ppat.1002803.s005.tif]

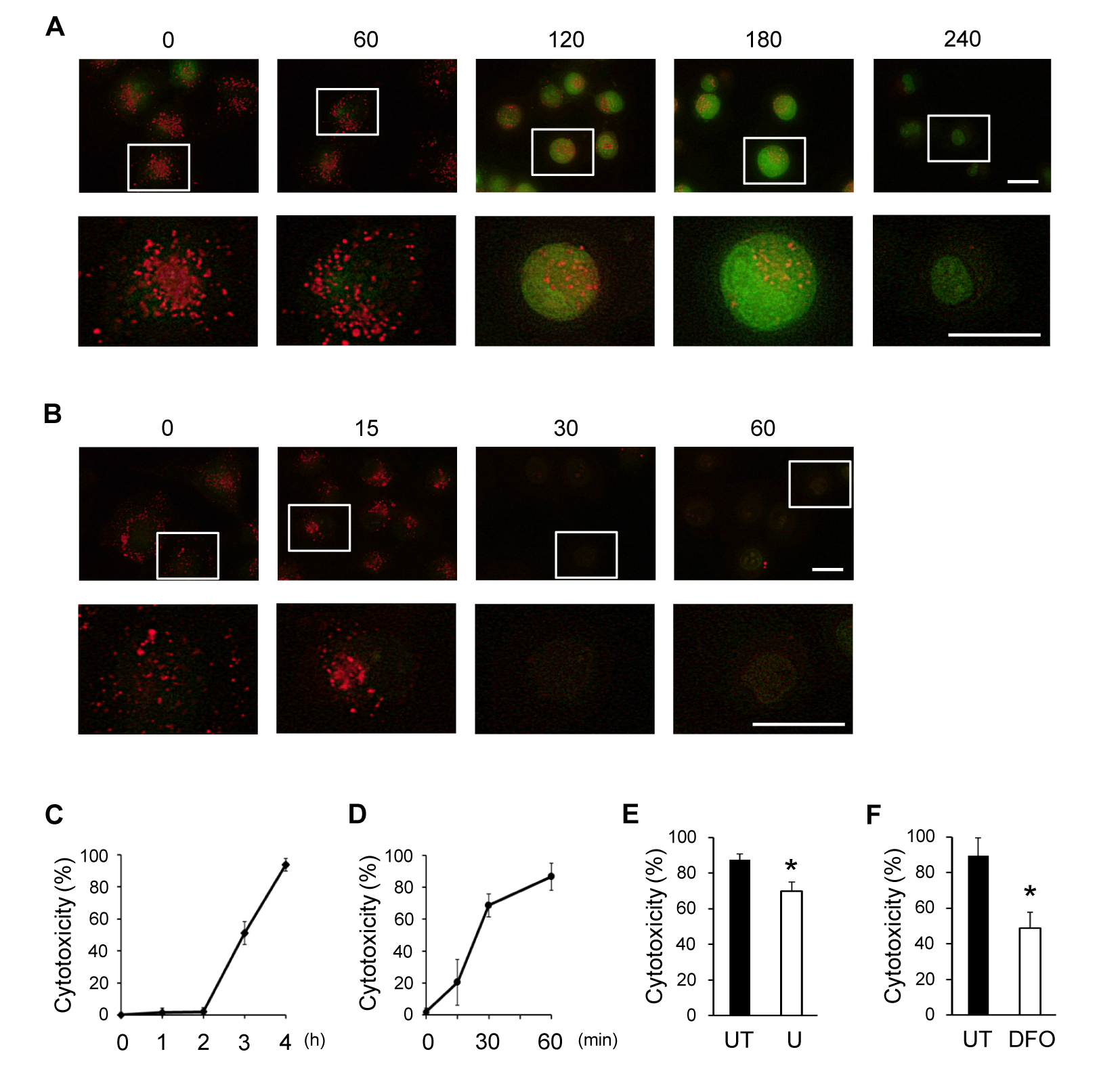

Supplement: Figure S6 — Time-course analysis of HeLa cells infected with V. parahaemolyticus or treated with TDH. (A, B) AO relocation assay in HeLa cells infected with V. parahaemolyticus POR-3 or treated with 100 µg ml−1 TDH toxin for the indicated times (min). Representative high magnifications of the areas indicated by white boxes in the top panels are shown in the bottom panels. Scale bar, 20 µm. (C, D) The cytotoxicity of HeLa cells infected with V. parahaemolyticus POR-3 (C) or treated with TDH (D) for the indicated times was evaluated by the LDH release assay. (E, F) The effects of lysosomal membrane stabilizers on the cytotoxicity induced by V. parahaemolyticus. HeLa cells were left untreated (UT) or were treated with U18666A (0.5 µg ml−1, 48 h) or deferoxamine (DFO; 10 µM, 24 h), and infected with POR-3 for 4 h. The cytotoxicity was determined using the LDH release assay. *P<0.01. (TIF) [file ppat.1002803.s006.tif]
