# Supplementary material for: A Cytotoxic Type III Secretion Effector of Vibrio parahaemolyticus Targets Vacuolar H+-ATPase Subunit c and Ruptures Host Cell Lysosomes
Source: PLoS Pathog. 2012 Jul 19;8(7):e1002803. doi: 10.1371/journal.ppat.1002803 (PMC3400558; doi:10.1371/journal.ppat.1002803)
Supplement: Table S1 — The plasmids and primers used in this study. (DOC) [file ppat.1002803.s007.doc]

**Table S1.** **The plasmids and primers used in this study.**

| **Plasmids** | **Descriptions** | **References** |
| --- | --- | --- |
| pSA19CP-MCS | Complement vector for *V. parahaemolyticus* | [14] |
| pSA-VepA | pSA19CP-MCS encoding VepA | [14] |
| pSA-VepA∆C | pSA19CP-MCS encoding VepA (1-400) | This study |
| p426GAL | galactose inducible yeast vector | [9] |
| p426-VepA | p426GAL encoding VepA | This study |
| p426-VepA∆C | p426GAL encoding VepA∆C (1-400) | This study |
| p426-VopP | p426GAL encoding VopP | [9] |
| p426-VopT | p426GAL encoding VopT | [9] |
| pEGFP-C1 | N-terminal EGFP fusion vector | Clontech |
| pEGFP-VepA | pEGFP-C1 encoding VepA | This study |
| pEGFP-VepA∆C | pEGFP-C1 encoding VepA∆C (1-400) | This study |
| pEGFP-N1 | C-terminal EGFP fusion vector | Clontech |
| pATP6V0C-Flag | pEGFP-N1 encoding ATP6V0C with  C-terminal Flag and stop codon | This study |
| pET30a | Expression vector for *E. coli* | Novagen |
| pET30a-VepA | pET30a encoding VepA | This study |
| pET30a-VepA∆C | pET30a encoding VepA (1-400) | This study |
|  |  |  |
| **Primers** | **Sequences (5' to 3')** | **Notes** |
| ATP6V0C-Flag-F | gaattcatgtccgagtccaagagcagcggccccg | ATP6V0C-Flag |
| ATP6V0C-Flag-R | gtcgacctacttgtcatcgtcgtccttgtagtcctttg tggagaggatgagggcgacg | ATP6V0C-Flag |
| VepA-F | gaattctatggtgaatacaacgcaaaaaatc | Cloning for VepA |
| VepA-R | gtcgacttaaatccagccttcggctaag | Cloning for VepA |
| VepAΔC-R | gtcgacttattcatcgctcagtttaagcag | Cloning for VepA∆C |
| V0C (31-80)-F | gaattctgcctatggcacagccaagag | Cloning for Vc1 |
| V0C (31-80)-R | gtcgacctacagggagttggcgatgagga | Cloning for Vc1 |
| V0C (111-155)-F | gaattctggcatcgtgggggacgctgg | Cloning for Vc2 |
| V0C (111-155)-R | gtcgacctactttgtggagaggatga | Cloning for Vc2 |
| VepA∆C-F | ctgagcgatgaataaatgccaagtcag | Used for pSA-VepA∆C construct |
| VepA∆C-R | ctgacttggcatttattcatcgctcag | Used for pSA-VepA∆C construct |
| U1 | gatgtccacgaggtctct | Amplification of tag regions |
| D1 | cggtgtcggtctcgtag | Amplification of tag regions |
| KanB | ctgcagcgaggagccgtaat | Sequencing for up-tag |
| KanC | cctcgacatcatctgcccagat | Sequencing for down-tag |
